# Ununited Fractures

**Published:** 1855-02

**Authors:** 


*Ununited Fractures.*—Dr. Henry H. Smith has republished from the American Journal of Medical Sciences, his paper on Ununited Fractures. His treatment consists in placing the limb in an immovable apparatus for a series of months, or rather, in an artificial limb, allowing the patient to get about, and thus hasten, by exercise, &c., the union of the fracture.

No surgeon, nowadays, is allowed to originate anything. We put in a reclamation. "On or about" the first of October, 1845, we saw an artificial limb in the possession of Prof. Geo. McCook, now of Pittsburg, Pa., intended for the treatment of an ununited tibia, and very nearly resembling Dr. Smith's apparatus in its style of manufacture, and precisely in the principle and objects of its application. H.

